# Three-dimensional magnetic cloak working from d.c. to 250 kHz

**DOI:** 10.1038/ncomms9931

**Published:** 2015-11-24

**Authors:** Jianfei Zhu, Wei Jiang, Yichao Liu, Ge Yin, Jun Yuan, Sailing He, Yungui Ma

**Affiliations:** 1 State Key Lab of Modern Optical Instrumentation, Centre for Optical and Electromagnetic Research, College of Optical Science and Engineering, Zhejiang University, Hangzhou 310058, China; 2 Department of Electromagnetic Engineering, School of Electrical Engineering, Royal Institute of Technology, S-100 44 Stockholm, Sweden; 3 ZJU-SCNU Joint Research Center of Photonics, South China Academy of Advanced Optoelectronics, South China Normal University, 510006 Guangzhou, China

## Abstract

Invisible cloaking is one of the major outcomes of the metamaterial research, but the practical potential, in particular for high frequencies (for example, microwave to visible light), is fatally challenged by the complex material properties they usually demand. On the other hand, it will be advantageous and also technologically instrumental to design cloaking devices for applications at low frequencies where electromagnetic components are favourably uncoupled. In this work, we vastly develop the bilayer approach to create a three-dimensional magnetic cloak able to work in both static and dynamic fields. Under the quasi-static approximation, we demonstrate a perfect magnetic cloaking device with a large frequency band from 0 to 250 kHz. The practical potential of our device is experimentally verified by using a commercial metal detector, which may lead us to having a real cloaking application where the dynamic magnetic field can be manipulated in desired ways.

## Abstract (web summary)

The development of invisibility cloaks which function at low frequencies are of practical importance, especially for magnetic fields involved in modern technologies. Here, Zhu *et al*. develop the bilayer approach to create a three-dimensional magnetic cloak able to work in both static and dynamic fields.

## Graphical abstract

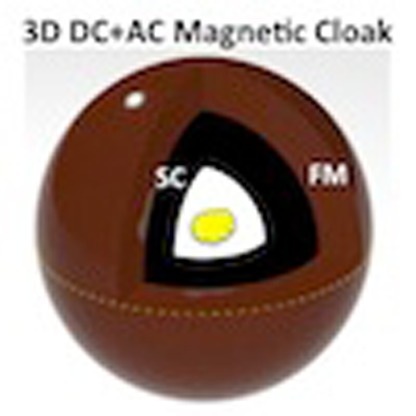

In the last decade, invisible cloaking or hiding things from detection has gained extensive research enthusiasm in the field of metamaterials and pertinent communities since the proposal of the original conceptions^1^,^2^. Modern transformation optics techniques have been developed to configure and modulate the cloaking devices associated with the development of complex metamaterials with extreme electromagnetic properties^3^,^4^,^5^,^6^,^7^,^8^. To improve the practical feasibility, various modified versions for electromagnetic cloaks have been proposed and demonstrated, among which the most explored are the quasi-conformal^9^,^10^,^11^,^12^,^13^,^14^,^15^,^16^ and (later) the bilinearly transformed cloaks^17^,^18^,^19^. These modified transformation optics approaches allow the usage of weakly anisotropic or isotropic artificial/natural materials to fabricate the appliances, which greatly improves the loss and bandwidth properties. However, individually they have their own inherit limits either in incident angles or in polarization states^20^,^21^. Precise fabrication of these devices with acceptable reproducibility and cost is still very challenging, especially for those aimed to work at high frequencies (that is, microwave to terahertz or visible light). These hurdles need to be overcome in the future with smarter designs or more accessible cloaking algorithms.

On the other hand, invisible cloaks for single components of electromagnetic fields at d.c. or low frequencies are also of practical importance^22^,^23^,^24^,^25^,^26^,^27^,^28^,^29^, especially for magnetic fields that are universally involved in numerous modern facilities and technologies^22^,^23^,^24^,^27^,^28^,^29^. Superconducting (SC) materials with static perfect diamagnetism (*μ*=0) play a key role in designing a d.c. magnetic cloak^22^,^30^,^31^,^32^. Technically though, it won't be easy to fabricate a magnetic cloak obtained from transformation optics even by a composite of SC and ferromagnetic (FM) materials. To solve this issue, a promising bilayer approach was initially proposed by Sanchez's group to build a static magnetic cloak on the basis of uncoupled Maxwell's equations, that is, Laplace's equation^23^. Although the theory of this bilayer approach starts from a uniform external field, it is found to still be functional in certain inhomogeneous environments, even like a magnetic dipolar field^28^,^32^. Because of its simplicity and efficiency, the bilayer cloak approach has attracted considerable attention and has been quickly applied to many other parallel systems mainly controlled by diffusion equations, such as thermal flux^33^,^34^, electric current^35^, ions^36^ and diffusion light^37^. One application in particular is an electro-thermal bifunctional cloaking device, which was recently demonstrated by the authors on the basis of this bilayer architecture^38^.

These experimental progresses enabled by the bilayer approach may bring us more confidence to pursue a practical cloaking device specifically for applications at low frequencies where the quasi-static approximation is valid^39^. Using a cylinder made of SC and FM composites, Sanchez's group has demonstrated the possibility to realize a static magnetic cloak first in a two-dimensional field^23^. A real practical device is generally required to work in a three-dimensional (3D) space. More importantly, dynamical functionalities with selectable operation bands are highly desired, especially corresponding to those used by magnetic field induction technologies that are commonly relied on to uncover the hidden or underground magnetic/metallic objects^40^. In another paper^27^, Sanchez's group made a first attempt in this aspect and showed a limited quasi-static magnetic cloaking effect from 0 to 144 Hz utilizing a similar two-dimensional cloaking structure. Quite recently, the same group extended this bilayer structure to demonstrate a 3D invisible wormhole device that can transfer d.c. magnetic field^32^. The sample fabrication will become significantly more difficult in three dimensions because the usual flat FM or SC sheets cannot be applied here anymore for spherical topologies. Dynamic field applications will also raise the requirements on material properties such as nonlinear hysteresis and/or conductive loss of the FM component and the transport capability or critical field strength of the SC component. Besides these material issues, there is a general question of whether the SC component can still be as effective as a zero-permeability entity in time-varying fields where inhomogeneous vectorial Helmholtz equations have to apply.

Regarding these critical issues, in this work we vastly develop the bilayer approach to pursue a magnetic cloak operational in a 3D quasi-static field by optimizing material properties. In contrast with the metallic alloys used before^23^,^27^,^28^,^32^, resistive high-quality ferrite is employed here to remove the eddy-current loss and more importantly to acquire a linear magnetic response in a relatively broad field range. For the magnetized sample, a nearly flat permeability spectrum is achieved in a frequency band from d.c. to hundreds of kilohertz. The SC component we use is carefully manufactured from single-crystal yttrium barium copper oxide (YBCO) cylinders, whose bulk and single-crystal features could exclude many possible negative material issues related to inductive loss. With such a bilayer structure, here we experimentally show a perfect 3D magnetic cloak working from d.c. to a maximum measurement frequency of 250 kHz, which covers the operation bands of nearly all EMI appliances. The application potential to hide objects in a real field is also examined by using a commercial metal detector.

## Results

### Sample design and fabrication

**Figure 1:**
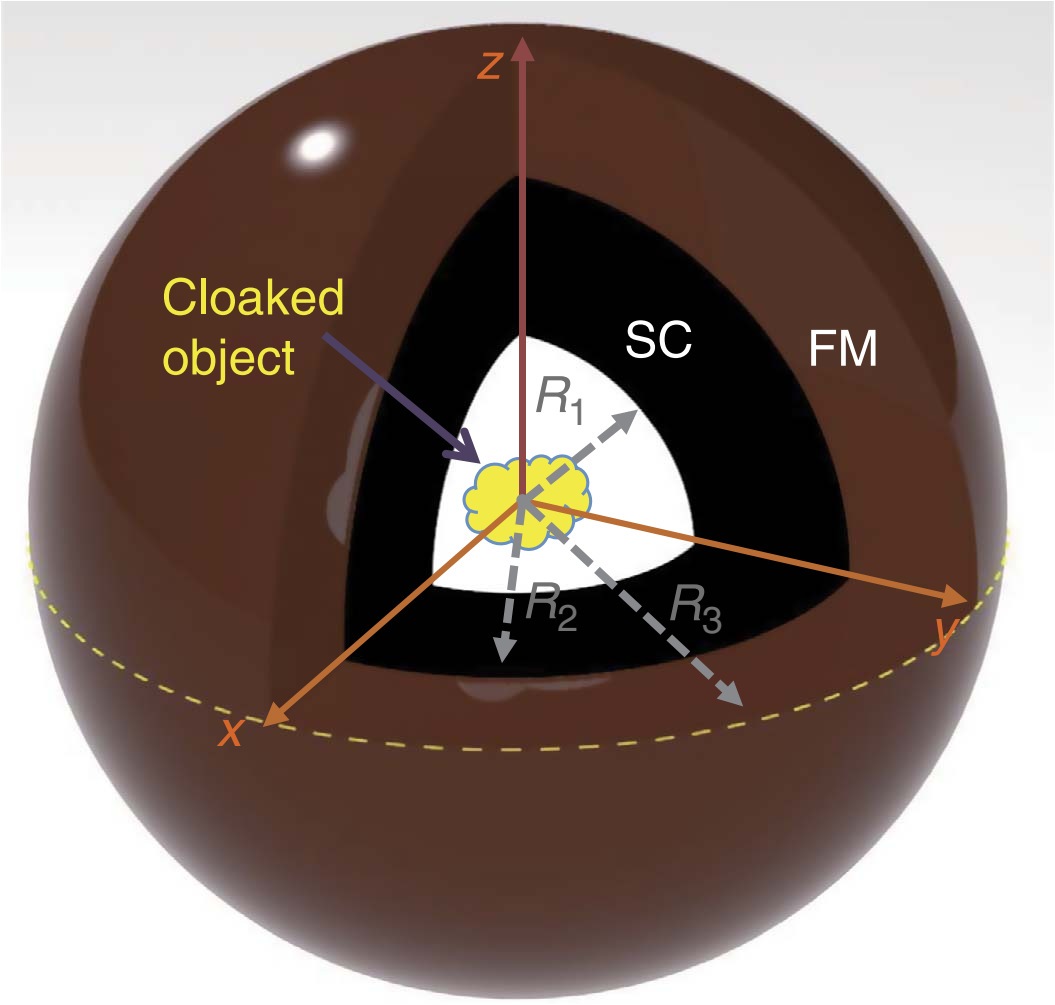
Schematic of the device. The bilayer structure consists of an inner SC shell (*R*_1_≤*r*<*R*_2_), shown in black, and an outer FM shell (*R*_2_≤*r*≤*R*_3_), shown in brown. The two identical halves of their components touch each other in the *xy* plane as indicated by the yellow dashed circle. The cloaked object (shown in yellow) is placed inside the cloaking region (*r*<*R*_1_), shown in white. In our measurement, the *c* axis of the YBCO's unit cell in the SC shell is defined along the *z* axis, and the *ab* lattice plane is parallel to the *xy* coordinate plane. In our experiment, we select *R*_3_=1.5 *R*_2_=15 mm, which leads to *μ*_FM_=1.63.

Figure 1 provides a schematic of the bilayer structure consisting of the SC inner shell (black) and FM outer shell (brown) in a non-magnetic background. Each shell consists of two firmly connected identical halves touching each other in the *xy* plane. The SC shell (inner radius *R*_1_ and outer radius *R*_2_) was machined and etched from two YBCO single-crystal cylinders. In Cartesian coordinates, the *z* axis is defined along the *c* axis of the YBCO's unit cell and the *xy* plane is parallel with the *ab* lattice plane. In this manner, the maximum applicable magnetic field is different along the *z* axis than a direction in the *xy* plane due to the material anisotropy^41^. The FM shell of outer radius *R*_3_ is a composite of NiZn soft ferrite powders and paraffin matrix by a proper weight ratio. The fabrication details can be found in the Methods section. Assuming a uniform static external field and a perfect SC shell (skin depth or London penetration depth is in the sub-micron scale^41^), the FM component required by an ideal 3D magnetic cloak should have a permeability of (see ref. 23 and also Supplementary Note 1)


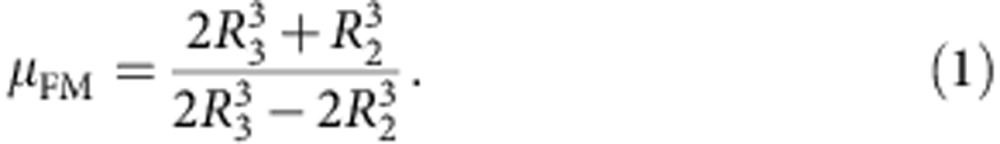


By this bilayer structure, any magnetic or conductive object (yellow) placed inside the cloaked region (white) should be magnetically invisible (or undetectable) to a nearby observer^29^. In practice, we select *R*_3_=1.5 *R*_2_=15 mm so that the desired *μ*_FM_ is equal to a small number (1.63). The relatively large thickness and low permeability of the FM shell is helpful in minimizing the demagnetization shape effect and thus improves the isotropic response of the FM shell. The diluted magnetic ingredient in the composite also helps control the magnetic residual loss.

### Simulation results

**Figure 2:**
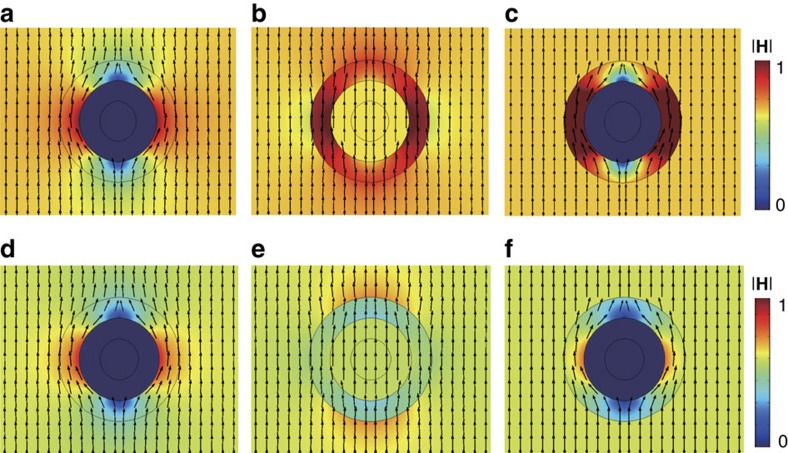
Simulation results. (**a**–**c**) The magnetic field intensity profile for the static field. (**d**–**f**) The magnetic field intensity profile for the dynamic field at 25 kHz. The samples are made of SC material only (**a**,**d**), FM material only (**b**,**e**) and the bilayer composite (**c**,**f**), respectively. Different colours represent the absolute value of the local magnetic field normalized by the largest value and the black arrow lines represent their directions. For both static and dynamic cases, the bilayer sample shows no perturbation to the external magnetic field, and thus a perfect 3D cloaking is realized under the quasi-static approximation.

Numerical simulation is first carried out to examine the cloaking performance of the proposed device. The modelling details for static and dynamic simulations can be found in the Methods section. Figure 2a–c shows the modulus profiles of static magnetic field in the *xz* plane for the samples made of SC only, FM only and the bilayer composite (SC+FM), respectively. The normalized strength of the magnetic field is represented by different colours, and the field direction is represented by the black arrow lines. It is apparent that the magnetic force lines on the top of the sample are expelled by the sole SC shell (Fig. 2a) and concentrated by the sole FM shell (Fig. 2b), while these perturbations are completely cancelled by their proper combination (Fig. 2c). These results are more quantitatively illustrated by the field change curves calculated along one straight line at a 5-mm distance above the sample in Supplementary Fig. 1a.

To simulate the dynamic response, which means the behaviour of a sample in the time-harmonic oscillating field in this paper, we assume our single-crystal YBCO bulk has a conductivity 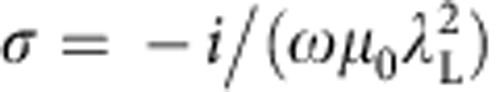 S m^−1^ at 77 K determined by the normal-state eddy current^42^,^43^, where 
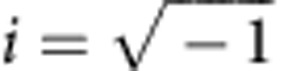
, *ω* is the angular frequency, *μ*_0_ is permeability and *λ*_L_ is the London penetration depth. As a perfect conductor, SC's dynamic behaviour is dominantly decided by its electric properties, and the influence of permeability is less important and practically could be neglected. Figure 2d–f shows the simulated magnetic field amplitude profiles in the *xz* plane at 25 kHz for the devices consisting of different material components. The magnitude of the local magnetic field, normalized by its largest value, is represented by different colours and the orientation at the moment that the consine-waveform external field has zero phase is denoted by the black arrow lines. They exhibit similar field interaction patterns as their static counterparts shown in the top panel. These dynamic field features are nearly independent of the simulation frequency from 100 Hz to 1 MHz. Under the quasi-static approximation, these frequency behaviours are quite reasonable because Maxwell's equation outside the SC region could still be effectively approximated by a Laplace equation, and the infinitely large conductivity of a superconductor will take over the static Meissner effect to screen out the oscillating electromagnetic field. The latter effect makes the SC component still act as a perfect diamagnetic entity. Consequently, at low frequencies, an SC or FM shell alone or their combination will almost replicate their static field behaviours, provided that their material parameters are constant at different frequencies. The perfect dynamic cloaking performance is evidenced by the magnetic field intensity distribution in Fig. 2f. A more quantitative analysis is given in Supplementary Fig. 1b by plotting the relative change of the nearby field.

### Cloaking measurement in static magnetic fields

**Figure 3:**
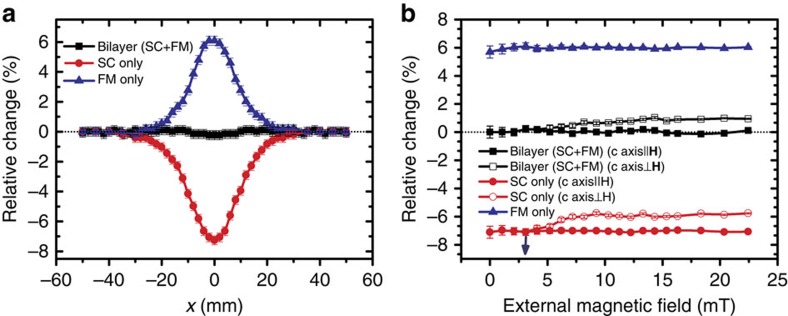
Measurement results for static field. (**a**) The relative change of the *z*-component magnetic field measured for the bilayer sample (black squares) and two references with SC (red circles) and FM (blue triangles) shells only along the straight line at *z*=*R*_3_+5 mm in the *xz* plane. In the measurement, a uniform external magnetic field of 2.5 mT is applied along the *z* axis. (**b**) The relative change of the magnetic field as a function of the strength of the external magnetic field applied along the *z* axis. The sample of FM shell only is measured once, while the rest two samples are measured twice with the *c* axis of the YBCO unit cell parallel to the external magnetic field (*c* axis||**H**) and perpendicular to it (after rotating the sample around the *x* axis by 90°) (*c* axis⊥**H**), respectively. The anisotropic property of the lower critical field for YBCO single crystals leads to the ‘splitting' of the measured in-plane relative change at about 2.8 mT. Our Hall sensor has a voltage resolution at 0.1 μV. The error bars in **a** and **b** are obtained by dividing this value with the measured sample voltage.

For the static field measurement, we use a pair of commercial Helmholtz coils that can provide a maximum magnetic field of 25 mT. All the samples are first cooled down in a liquid nitrogen bath (77 K) with no external magnetic field before measurement. To check the field perturbation, we scan the *z*-component distribution of magnetic field in the *xz* plane along the straight line *z*=*R*_3_+*h*, where *h* is a distance parameter fixed at 5 mm for both d.c. and dynamic field measurements. Note this *h* value is selected to be one-sixth of the diameter of the whole device to make sure that the near-field features are characterized and on the other hand it is also a proper distance convenient for experimental operation. We also experimentally confirmed that smaller distance gave no obvious difference on the near-field cloaking effect. The relative change percentage, defined by 100(*V*_s_−*V*_0_)/*V*_0_ with *V*_s_ and *V*_0_ representing the measured Hall voltages with and without the sample, respectively, is used in our descriptions to evaluate the field variation due to the introduction of an object. The relative change for ideal cloaking should be zero. Figure 3a plots the relative change curves of the *z*-component magnetic field measured for the bilayer sample (squared black) and the two references with an SC (dotted red) or FM (triangle blue) shell only. In the measurement, a uniform external field of 2.5 mT is applied along the *z* axis. The concentrating or expelling disturbance to the nearby magnetic field is clearly observed due to the existence of the FM or SC shell, respectively. Note as a near-field effect this influence manifests only within a deviation distance of 35 mm, which agrees well with the simulation given in Supplementary Fig. 1a. In contrast, the relative field change caused by the bilayer sample is substantially suppressed and the measured amplitude fluctuates around null (dashed line), with absolute values within an uncertainty error of 0.3% of our experiment system. Because the sample has a rotational symmetry, similar field patterns could be expected in other positions. Therefore, it can be concluded that our bilayer structure gives a reasonably good realization of a d.c. magnetic field cloaking effect in 3D space.

For practical applications with different purposes, the field strength tolerance is a very important factor in evaluating the capability and potential of a real cloak. In this aspect, the linear response capability of the FM component and the maximum critical field for the SC component need to be carefully examined. Regarding the first issue, NiZn spinel ferrites with relatively large magnetocrystalline anisotropic energy among soft magnetic materials have been employed in this work to acquire a relatively high-field linear magnetization property. Supplementary Fig. 2a plots the magnetization hysteresis loop of our FM composite at a maximum field of 1 T at 77 K. The inset for a zoom-in minor loop shows the magnetic composite has a good linear magnetization behaviour at least up to 20 mT, which could be large enough for most low-field applications. This linear characteristic is important for the current amplitude and frequency-based field perturbation measurement^29^.

Figure 3b gives the relative changes of the *z*-component magnetic field as a function of the external field strength measured at a fixed point *z*_0_=*R*_3_+*h* on the *z* axis for the structures with different material compositions. For the sole FM shell, the relative field change (blue) is nearly constant at ∼6%, which is consistent with the linear magnetization loop given in the inset of Supplementary Fig. 2a. For the samples with a SC shell, we repeat the measurement by applying the external magnetic field **H** parallel and perpendicular (after rotating the sample around the *x* axis by 90°) to the *c* axis of the YBCO unit cell, respectively. For the SC and bilayer samples, the relative field change (red) measured along these two different directions starts to ‘split' at a field near 2.8 mT. As a typical type II superconductor, YBCO's lower critical field in the *ab* plane (∼2.5 mT) has been found to be nearly 10 times smaller than that along the *c* axis (∼26 mT) at 77 K (ref. 39). Below this lower limit, it works in the Meissner state as a perfect diamagnet and turns into a mixed state above this limit where the field starts to penetrate the SC body. It imposes our magnetic cloak an upper limit for the working field (not more than 2.5 mT) for general applications. However, this limit could be improved if the external field direction is known in advance.

### Cloaking measurement in dynamic magnetic fields

**Figure 4:**
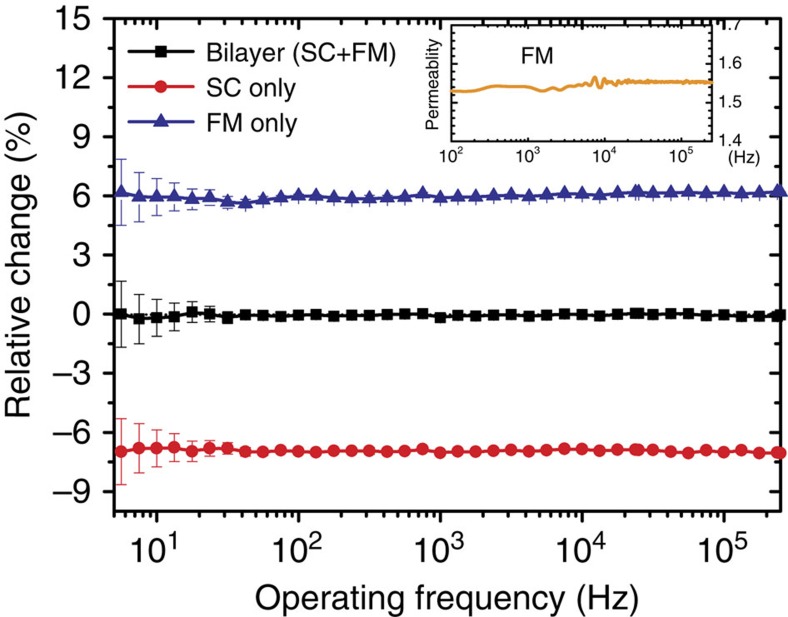
Measurement results for dynamic field. This displays the measured relative change of the *z*-component magnetic field intensity at a fixed point *x*=*y*=0 and *z*=*R*_3_+5 mm when the operating frequency varies from 5 Hz to 250 kHz. Here the magnetic field amplitude we apply is smaller than 1 mT. The measured induction voltage signal is nearly a purely imaginary number due to the low loss features of our ingredient materials used for the device. The uncertainty error arising mainly from the background noise becomes manifest at lower frequencies, especially below 50 Hz, because the measured signal decreases (and gets closer to the noise level) as the measurement frequency decreases. The samples of FM and SC shells only have nearly constant values around 6 and 7%, respectively, while the bilayer structure has almost zero relative change in the measurement band. The inset plots the real permeability spectrum of the FM material measured at room temperature, from 100 Hz to 250 kHz. The diluted composite has a negligible loss tangent (<0.01). The permeability spectrum at 77 K is estimated by multiplying by a factor proportional to the increase of the saturation magnetization due to the reduction in temperature. The minimum resolvable voltage of our lock-in amplifier is dependent on the operation frequency. The error bars in the figure are calculated by dividing the resolution with the measured sample voltage.

For dynamic field measurement, we use a pair of custom-made Helmholtz coils with proper inductance. A Stanford signal generator is used to excite the coils, and the *z*-component magnetic field near the sample is inductively measured through a small copper loop connected to a lock-in amplifier. The field strength exposed to the sample is <1 mT, and the measurement frequency varies from 5 Hz to 250 kHz to suit the band of our lock-in amplifier. Figure 4 gives the measured relative change of the *z*-component magnetic field intensity at a fixed point *x*=*y*=0 and *z*_0_=*R*_3_+*h* (*h*=5 mm) for various samples. For the dynamic case, the magnitude of the induced voltage has been used to evaluate the relative change of the magnetic field component. As discussed later, the ingredient FM and SC materials we used have very low losses in the operation state. The induced complex voltage signals are nearly pure imaginary numbers in the measurement frequency band, that is, a phase difference around 90°. (with ±0.5° fluctuation) from the source current of the exciting coil. The influence of the real part of the measured signal is practically neglected in our consideration. From the curves, we see that the dynamic responses of these samples are nearly independent of the operating frequency. The inset of Fig. 4 shows the measured room temperature permeability spectrum of our FM composite from 100 Hz to 250 kHz. A nearly constant permeability around 1.54 is observed, which is essential for the large bandwidth of our cloaking device. Permeability at the working temperature (77 K) is estimated by multiplying the ratio of the saturation magnetization measured at these two different temperatures. Fine tuning of the composition in experiment is carried out till the lowest field perturbation is achieved. The measured relative changes of the *z*-component magnetic field intensity for the SC and FM shells only remain at about 6% and 7%, respectively, while that for the bilayer sample is desirably diminished to nearly zero (absolute amplitude <0.2%). Note the measurement at low frequencies (<50 Hz) has an increased uncertainty error due to the fact that the measured induction signal is proportional to the operation frequency and the influence of the background noise becomes more obvious at lower frequencies. However it will not impact the conclusion that the measured results verify the dynamic cloaking performance of our device from low frequency up to 250 kHz, where the quasi-static approximation works fairly well. In addition, we did check the field disturbance at places much closer to the sample surface, that is, at smaller *h*, and found the relative change of the *z*-component magnetic field increases slightly with fluctuation varying from 0.2% at *h*=5 mm to 0.3% at *h*=0. This also demonstrated the advantage of the 3D cloaking topology.

**Figure 5:**
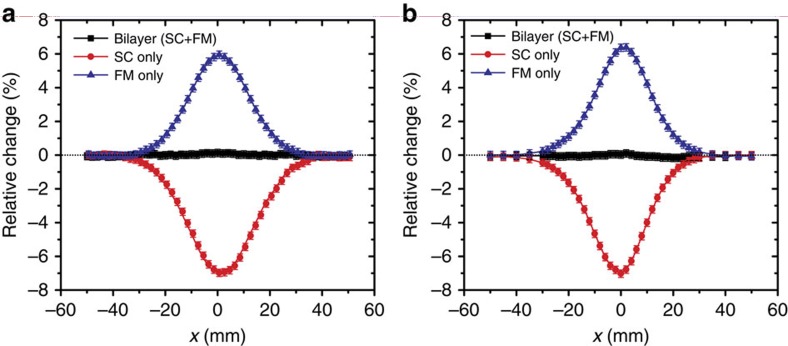
Measurement results for dynamic field at 25 kHz. The measurement is conducted in the *xz* plane for the *z*-component of the near-field magnetic field by scanning the prober along the line at *z*=*R*_3_+5 mm over the sample fixed at the origin (**a**) or moving the sample along the line at *z*=0 under the probe fixed at *x*=0 and *z*=*R*_3_+5 mm (**b**), respectively. In the second measurement, the magnetic field experienced by the samples has a relative strength change of about 4% due to the spatial inhomogeneous distribution of the excitation field. The two different measurements yield similar results, indicating the robustness and isotropic field response of our samples. Our lock-in amplifier has a voltage resolution of 0.2 μV at 25 kHz. The error bars in **a** and **b** are obtained by dividing this value with the measured sample voltage.

To get a full picture about the perturbation to the exciting field, we measure the relative change of the *z*-component magnetic field in the *xz* plane at 25 kHz by two ways: one is scanning the probe along the line *z*=*R*_3_+*h* over the sample fixed at the origin and the other is moving the sample along the line *z*=0 under the probe fixed at the point *x*=0 and *z*=*R*_3_+*h*. These two measurements examine the capability of our bilayer sample to work in a uniform field and a space-varying field, respectively, and the results are given in Fig. 5a,b, respectively. The latter condition caused by the real field distribution of our finite exciting coils corresponds to a maximum relative incident field change of about 4% along the measured line. The disturbance to the external field by the SC or FM shell due to magnetic induction or moment is clearly observed. For the bilayer sample, the disturbance to the near field is substantially suppressed, and the measured relative change is smaller than 0.2%. These measured results are in good agreement with the simulations shown in Supplementary Fig. 1b.

So far we have confirmed the dynamic cloaking capability of our bilayer structure in time-harmonic uniform magnetic fields with frequencies varying from 0 to 250 kHz. Through a Fourier transform we may expect that the same device can also work in the time domain as well with an arbitrary time-varying field, for example, an impulse field. In addition, it has been shown that the performance of the bilayer cloak is robust against the exciting field distribution^28^,^32^, which is similarly verified in our electric–thermal bifunctional cloak also designed by the bilayer approach^38^. In the last experiment here, we check the time-space response of our sample by using a commercial metal probe scanner that detects a conductive object by perceiving the change of the EMI signal^44^. The scanner has a transmitter coil to generate the probing field at a working frequency of 25 kHz and a pickup coil to measure the field change. In experiment, it is held to sweep over the sample at a distance of about 2 cm and will respond to the electromagnetic signature of a conductive object in two manners, that is, flashing the indicator (green to red) and making a warning sound ‘beep'. The measured results are given in Supplementary Movies 1 and 2. Supplementary Movie 1 shows that the scanner can perceive the room temperature bilayer sample (middle) and also two nearby references, that is, a metallic badge (left) of Zhejiang University and an aluminium sphere (*R*=10 mm) coated by 5-mm-thick paraffin (right), as shown in Supplementary Fig. 3. After immersed in liquid nitrogen for several minutes (Supplementary Movie 2), our cooled bilayer sample successfully ‘cheats' the prober without triggering the alarms. In this case, our bilayer shell can hide a metallic object inside and make the whole structure transparent to dynamic magnetic fields. The exact voltage signals generated by the pickup coil are also measured by an oscilloscope with the results plotted in Supplementary Fig. 4. From the modulation of the signal amplitude, we could recognize an approaching uncovered metallic object but are unable to access the cloaked sample.

## Discussion

We have experimentally confirmed the functionality of the bilayer cloak for both d.c. and dynamic fields under the quasi-static approximation. This assumption is valid only if the implemented device has a perfect SC component with a negligible field penetration depth compared with the sample size. An inner shell made of a common metal that has a frequency-dependent penetration depth at our interested frequencies cannot mimic the behaviour of a perfect diamagnet to completely screen out the primary field^45^. Supplementary Fig. 5 shows that the cloaking effect disappears when the inner SC shell is replaced by a normal conductor, such as copper. For our bilayer structure, the FM and SC components could be regarded as two polarization-opposite dipoles that balance each other's influence and induce a magnetic transparency effect^46^. In principle, several microns could be thick enough for the single-crystal SC shell if its manufacture was feasible.

To obtain good magnetic cloaking performance, the precise fabrication of the FM component with the desired properties is another necessary condition. First, the permeability of the FM component has to be accurately realized. Supplementary Fig. 6 shows that small deviations in the permeability from the optimal value by 6% will change much of the overall cloaking effect. Experimentally we reach the optimal value by gradually tuning the composition of the FM component around an estimated composition. The linearity of permeability within 20 mT is achieved by employing relatively ‘hard' NiZn spinel ferrite. Another issue for dynamic cloaking is the magnetic loss arising from both hysterical and residual losses for our insulating composite. In the experiment, the magnetic field amplitude we apply is <1 mT and under this condition, the magnetization switching around the remnant state is mostly a reversible process, and the residual loss due to the phase lag of magnetization rotation will be the dominant loss^47^. However, it won't be a serious problem in our bilayer structure because the ferrite constituent is vastly diluted by the paraffin matrix, which leads to a very small averaged imaginary permeability (<0.02), as shown in Supplementary Fig. 2b. For strong external fields, the weight of the hysterical loss will increase as theoretically predicted by Rayleigh's law^23^. On the other hand, this magnetic nonlinear loss could be potentially controlled by adopting a lower loss material composed of isolated single-domain particles (that is, removed Barkhausen jump) such as Co_2_Z hexagonal ferrite^48^, whose higher anisotropy also helps enlarge the maximum field limit and the operation bandwidth as well. By the current architecture, the maximum working frequency of up to 1 MHz can be expected. Beyond this, the magnetic cloak effect will gradually disappear due to the permeability change of the FM component and also the failure of the quasi-static approximation.

## Conclusion

We have demonstrated a 3D quasi-static magnetic cloaking device able to work from d.c. to 250 kHz. Control of the electric and magnetic material losses is realized by selecting high-quality NiZn ferrite and single-crystal YBCO as the ingredients of the FM and SC components, respectively. An isotropic flat permeability with negligible residual loss is obtained from d.c. to hundreds of kilohertz for our FM composite. The perfect SC shell helps exclude many possible negative material issues related to inductive loss. In the experiment, the dynamic performance of our bilayered 3D cloak in a wide frequency band from d.c. to 250 kHz has been verified from the nearby field perturbation measurement, which is in good agreement with the simulations. The practical potential of our device in anti-probe technology for magnetic fields is also experimentally examined using a real metal detector. The same architecture may also find essential applications in places such as bio-experiments or magnetically sensitive equipment where perfect screening without distorting external magnetic fields is specifically required.

## Methods

### Simulation of the sample

Both static and quasi-static simulations are performed using the axial symmetry module of COMSOL multiphysics. The 3D sample is constructed with structural parameters *R*_1_=*R*_2_/2=*R*_3_/3=5 mm and materials parameters *μ*_FM_=1.63 for the FM component and 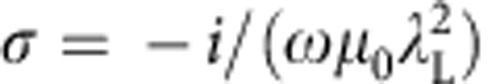 S m^−1^ for the single-crystal SC component^41^,^42^. We take a relative large penetration depth *λ*_L_=1 μm. A Laplace equation for the scalar magnetic potential is built to simulate the static field behaviours of the samples defined in a rectangular domain formed by two pairs of constant potential and magnetic insulation boundaries. For the dynamic simulation, an RF model with scattering boundary conditions has been based to mimic the quasi-static situation. A tall cylindrical current surface with height and radius far larger than the FM shell's radius *R*_3_ has been introduced to yield a well-defined uniform excitation environment for the sample. Since we only care about the near-field effect, the inaccuracies appearing in the far-field locations for low-frequency simulations could be practically excluded from our consideration.

### Fabrication of the sample

YBCO superconductor is used in our work due to its resilience at high temperatures. Two identical halves of the SC component with inner radius *R*_1_ and outer radius *R*_2_ are machined and chemically etched from two commercially bought YBCO cylinders (diameter=20 mm and height=10 mm). These two halves contact each other firmly in the *xy* plane (*z*=0) as shown in Fig. 1. Great care and patience are required during the fabrication process due to the very fragile nature of YBCO single crystals, which occupied most of the sample preparation time. The FM component with inner radius *R*_2_ and outer radius *R*_3_ is made of commercial NiZn ferrite powders and paraffin by using a standard moulding process. Magnetic hysteresis loops of the composite are measured at both room temperature and 77 K by a vibrating sample magnetometer (Lakeshore 7400). Reducing the temperature increases the measured saturation magnetization (Ms) by about 6%. The effective relative permeability of the composite as a function of the weight ratio of these two ingredients is first measured at room temperature by using an impedance analyser (Agilent 4294A) for donut-shaped samples. We estimate the permeability at 77 K by multiplying a factor from the change in Ms. Finer tuning of the weight ratio around a desired estimated value is conducted till we experimentally achieve an optimal cloaking effect. For the results reported here, the weight ratio of the powders to paraffin is 0.85, which corresponds to a room temperature relative permeability at about 1.54±0.01 from 100 Hz to 0.5 MHz. It is important to note that our magnetic sample for both static and dynamic experiments works in the linear portion of the hysteresis loop, and the non-zero remnant magnetization provides a self-biasing field.

### Measurement

For the static field measurement, the samples were placed at the centre of a pair of commercial Helmholtz coils (diameter=36 cm and spacing=14.5 cm) which can provide a maximum static magnetic field of 25 mT. The field direction is parallel to the *z* axis shown in Fig. 1. Before measurement, the samples were first cooled down with liquid nitrogen to 77 K with no external field. A low-temperature Hall sensor (Lakeshore HGCT-3020) was used to scan the *z*-component of the magnetic field along one straight line at *z*_0_=*R*_3_+5 mm in the *xz* plane, that is, above the sample by a 5-mm distance. High reproducibility of measurement data is guaranteed by utilizing a programmed step motor to move the prober. For the dynamic field measurement, a pair of Helmholtz coils (diameter=33 cm, spacing=11 cm and turns *N*=30) was built, which can satisfy the frequency band measurement, driven by a signal generator (Standard DS345). The *z*-component of the oscillating magnetic field near the sample is inductively measured by a ring prober (diameter=4 mm) made of 80-turn copper wire loops, connected to a lock-in amplifier (Signal Recovery 7270). The dynamic frequency is changed from 5 Hz to 250 kHz, decided by the lock-in amplifier we use. The voltage *V* used to represent the relative change of the dynamic field component is the magnitude (actually root mean square value) of the measured oscillating signal. Note that the real part of the measured voltage signal is always negligibly small compared with the imaginary part, and this is consistent with the low loss features of our ingredient materials used for the device. The induced signal is proportional to the frequency and suffers an increased measurement error at low frequencies due to the background noise. For the curves given in Fig. 5b, the prober is fixed at *x*=0 and *z*_0_=*R*_3_+5 mm and the samples are displaced along the *x* axis by ±50-mm intervals. In this manner, the samples experience a relative change of the incident field strength of about 4%. To examine the practical potential, a commercial handhold metal detector (Tianxu TX1001B) is used to scan the samples over a distance of roughly 2 cm. It consists of a pair of transmitting and receiving coils as well as an electronic circuit, and works at 25 kHz. When swept over a conductive or magnetic object, the EMI signal of the receiving coil will change in intensity and phase, which will then trigger the alarm (flashing the indicator and making a sound) if the change is larger than the threshold. The exact voltage signal of the receiving coil with respect to time is also measured by an Agilent (34410A) oscilloscope while keeping the scanner sweeping back and forth over the samples.

## Additional information

**How to cite this article:** Zhu, J. *et al.* Three-dimensional magnetic cloak working from d.c. to 250 kHz. *Nat. Commun.* 6:8931 doi: 10.1038/ncomms9931 (2015).

## Supplementary Material

Supplementary Information Supplementary Figures 1-6, Supplementary Note 1 and Supplementary References.

Supplementary Movie 1 Measurement of the bilayer sample (middle) BEFORE cooling by a metal prober. The two reference samples are a metallic badge of Zhejiang University in Chinese (left) and a paraffin coated aluminum ball (right).

Supplementary Movie 2 Measurement of the bilayer sample (middle) AFTER cooling by a metal prober. The two reference samples are a metallic badge of Zhejiang University in Chinese (left) and a paraffin coated aluminum ball (right).
