# Supplementary material for: Three-dimensional magnetic cloak working from d.c. to 250 kHz
Source: Nat Commun. 2015 Nov 24;6:8931. doi: 10.1038/ncomms9931 (PMC4696515; doi:10.1038/ncomms9931)
Supplement: Supplementary Information — Supplementary Figures 1-6, Supplementary Note 1 and Supplementary References. [file ncomms9931-s1.pdf]

(1) Cloaking effect for DC and time-harmonic fields

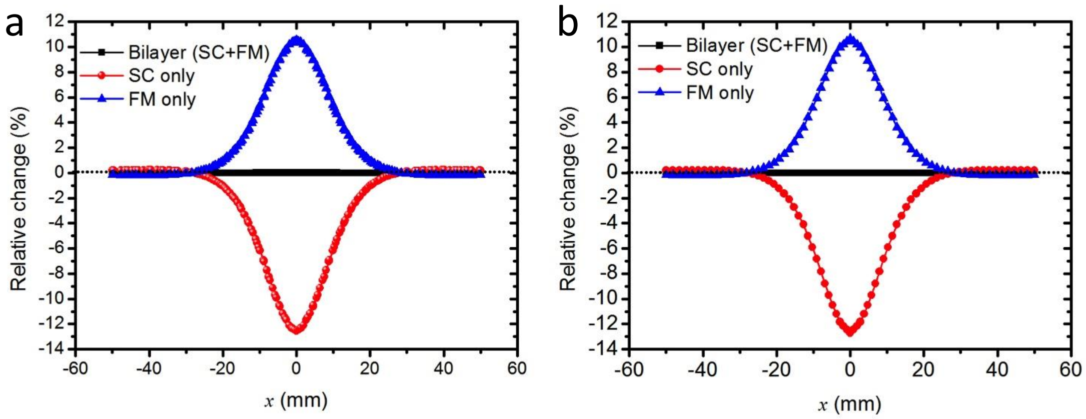

Supplementary Figure 1. Simulated relative change curve of magnetic field intensity. a, simulation for static magnetic field. **b**, simulation for time-harmonic field at 25 kHz. These field-intensity perturbation degrees are calculated along the straight-line at $z = R_3 + 5$mm in the *xz* plane. It can be seen that under the quasi-static approximation, the static and dynamic cases have the same field responses due to the introduction of the samples, i.e., FM shell only (blue triangles), SC shell only (red dots) and the bilayer composite (SC+FM) (black squares). It is clear that the FM (SC) shell concentrates (expels) the magnetic field lines above the sample, while the bilayer structure has zero perturbation to the uniform external field. Similar behaviors could be expected at different spatial positions. Thus, the bilayer structure realizes a perfect magnetic cloaking effect under the quasi-static condition.

(2) Magnetic properties of FM component

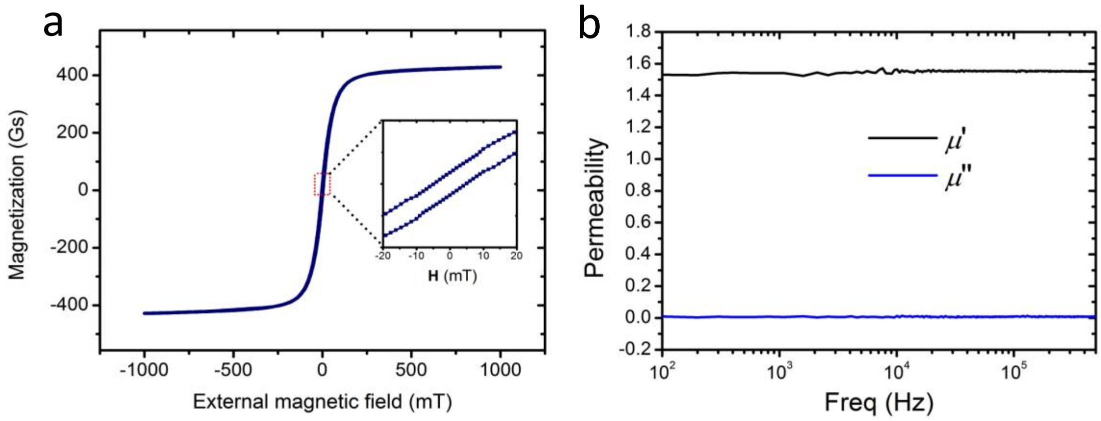

Supplementary Figure 2. Hysteresis loop and permeability spectra of the FM composite. a, the hysteresis loop measured by VSM at a maximum field of 1 T. The inset plots the zoom-in portion of hysteresis loop between ±20 mT. It is seen that the magnetization has a nearly linear switching behavior at lower fields. Minor loops with similar linear characteristics are measured at reduced maximum applied fields. The saturation magnetization of our composite is only one eighth of that for pure NiZn ferrite bulk[1]. But the effective permeability of the composite cannot be simply estimated from the reduction of the averaged magnetization because permeability is an extrinsic quantity dependent on both composition and structural parameters. Microscope measurement shows the NiZn powders we use have a grain size distribution from 1 to 3 um, which indicates demagnetization effect may take important role in the process of domain rotation or domain wall movement. **b,** the permeability spectra measured by an impedance analyzer. From 100 Hz to 0.5 MHz, our magnetic sample shows nearly flat permeability lines with the real part varying in 1.53-1.55 and the imaginary part is smaller than 0.02. The permeability value at 77 K is estimated by multiplying a factor (1.06) proportional to the incremental ratio of saturation magnetization under the assumption of negligible change of microstructures with temperature reduction. Fine tuning of the weight ratio of ferrite and paraffin is carried out till we experimentally achieve a best cloaking effect. The residual loss associated with magnetic rotation in our composite sample is not serious

in our interested frequency range as shown by the small imaginary permeability. The high resistance of the FM composite also excludes the electric conductive loss, which could be a serious issue to limit the working frequency for a metallic FM component[2,3].

(3) Measurement of metal detector

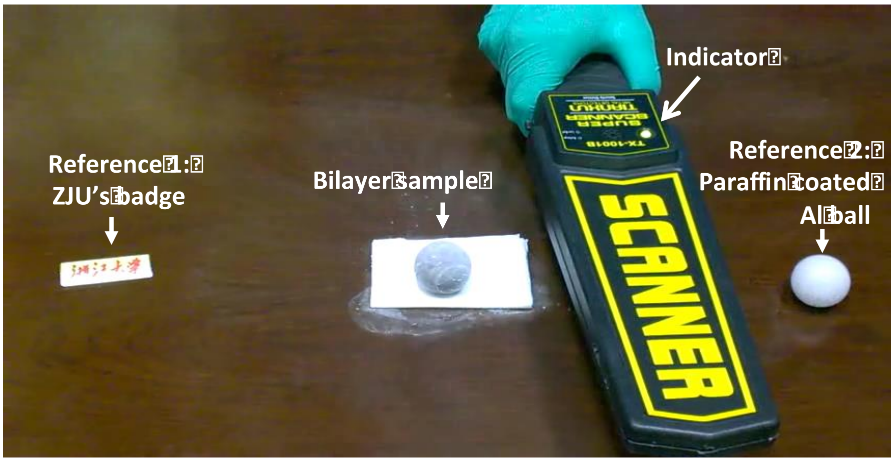

Supplementary Figure 3. Measurement of metal detector. The middle is the bilayer sample surrounded by smog due to its low temperature, the left and right are two references: a metallic Zhejiang University's badge in Chinese and a paraffin coated aluminum ball. The aluminum ball has the same radius as the SC component of the bilayer sample. The metal scanner is a commercial product widely used in the places like airport. For this one, it works at a fixed frequency 25 kHz. When swept over a metallic object, it will flash the indictor (green $\rightarrow$ red) and make a warning voice "beep". The supplementary Movies 1 and 2 give the measured results when the bilayer sample is before and after cooling down. The detector can recognize the room-temperature bilayer sample because it has a FM shell. After cooled down, the combination of SC and FM components is magnetically equivalent to a non-magnetic material and has no response to the external magnetic field.

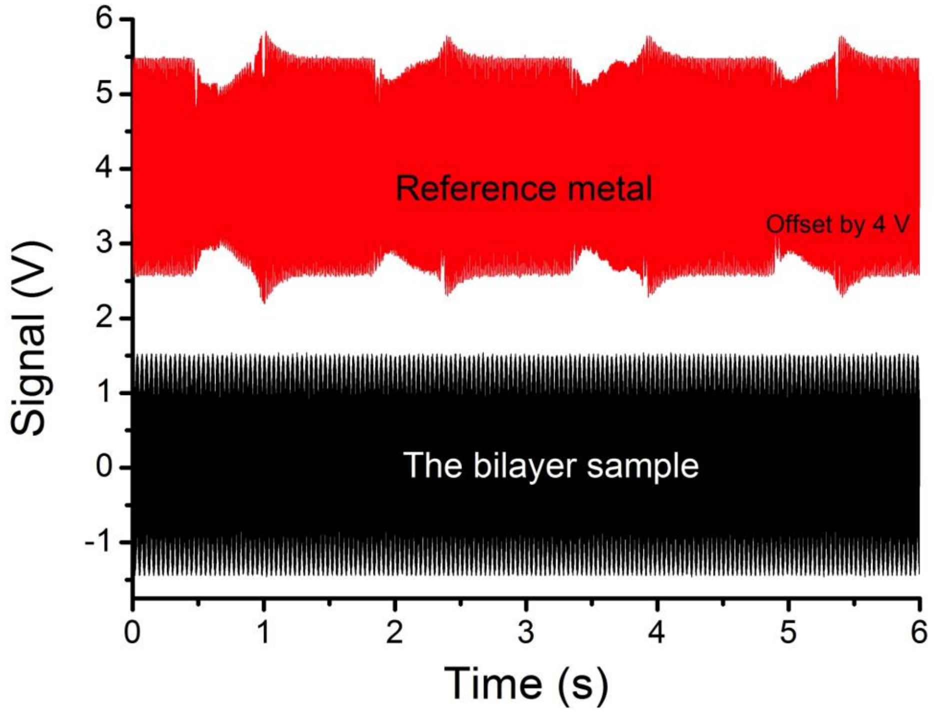

Supplementary Figure 4. Measured voltage signals in the receive coil of the metal detector. An oscilloscope is used to inspect the time-dependent voltage signal in the receive coil of the metal detector. The original signal is amplified by a built-in electronic circuit connected with the output of the receive coil. The above curve (red) is for a reference metal sample and the below (black) is the response when the detector is kept sweeping over the cooled bilayer sample back and forth. The red curve is offset upward by 4 V for comparison. It is clear that the receive coil or the detector can exactly recognize the existence of the uncovered metal object but has no response to the bilayer sample. It means our sample has reached the level for practical application.

(4) Influence of the conductivity of the SC component

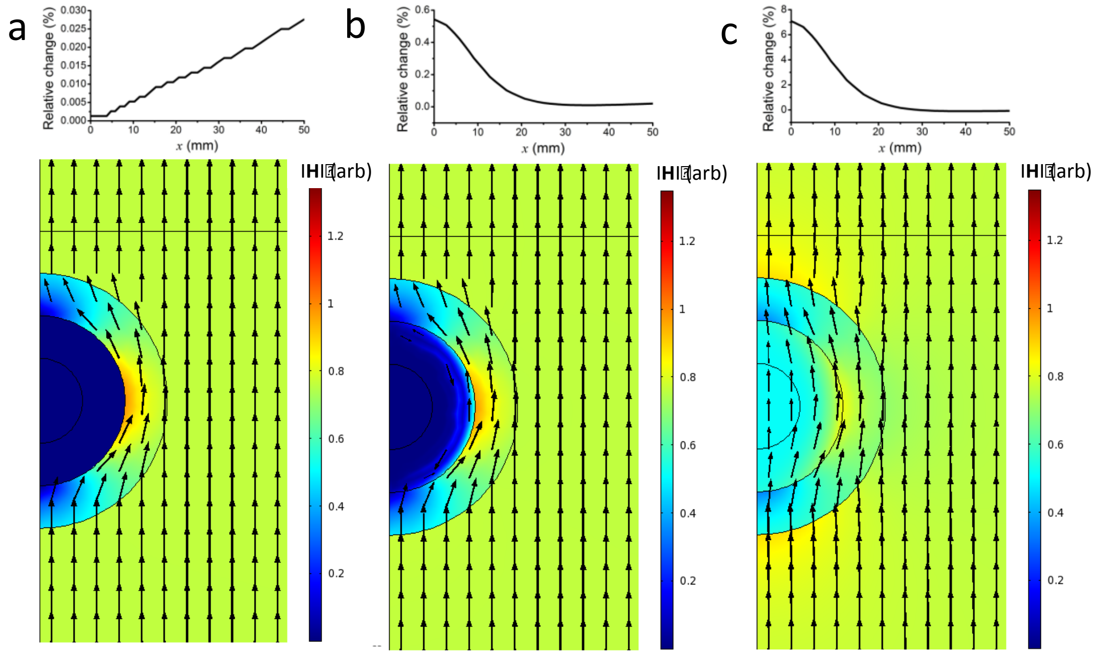

Supplementary Figure 5. Cloaking effect for different inner conductor. a, the ideal magnetic cloak with SC inner shell ($\sigma = -i5\times10^{12}$ S m^{-1}). **b,** the inner SC shell is replaced by a perfect conductor of $\sigma = 5\times10^{9}$ S m^{-1}. **c,** the inner SC shell is replaced by a good conductor of $\sigma = 5\times10^{7}$ S m^{-1}. The upper row gives the relative change of magnetic field intensity calculated along the black line shown in the bottom field patterns (half sides). The simulation is performed at 25 kHz. It can be seen that magnetic field starts to penetrate the inner conductor when the conductivity is reduced to 5×10^{9} S m^{-1}, but it still gives rise to good magnetic cloaking effect, as shown by the small relative change value (< 0.6%) in the top figure in (b). If using a good conductor, as shown in (c), the magnetic field totally penetrates the inner conductive shell at 25 kHz and the cloaking effect disappears. Therefore, a perfect conductor is necessary for dynamic magnetic cloaking.

(5) Influence of the permeability of the FM component

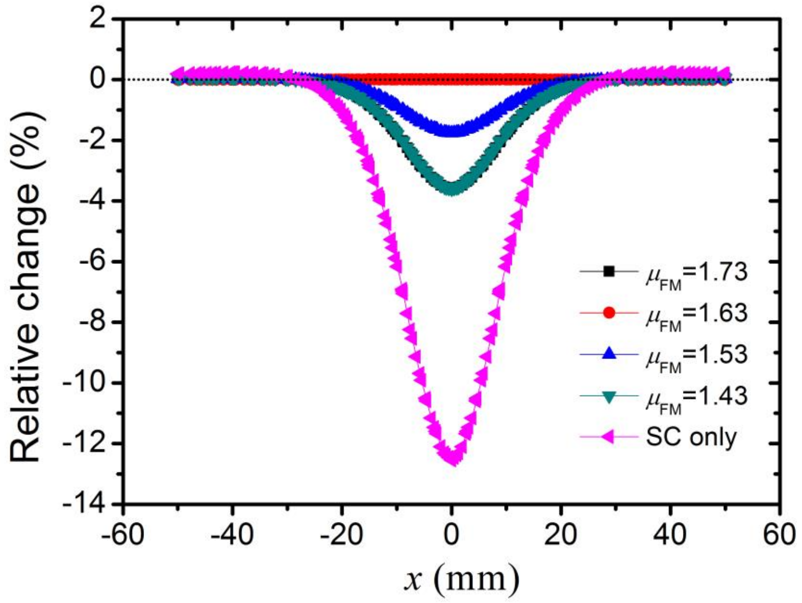

Supplementary Figure 6. Influence of the permeability of the FM layer. This figure plots the relative change of magnetic field intensity for the FM layer of different permeability values and a reference made of SC shell only. The desired value for an ideal magnetic cloak is 1.63 for the current structural parameters. It could be seen that change of permeability by 0.1 (or relative change of 6%) will obviously degrade the near-field cloaking effect. Thus accurate control of magnetic permeability is very important, which is experimentally realized in this work by finely tuning the weight ratio of the ferrite powders and the paraffin matrix. The influence of the imaginary permeability is neglected because it is very small (< 0.02) for our diluted magnetic composite.

SUPPLEMENTARY NOTE 1

For static magnetic field **H**, it could be described by a magnetic scalar potential φ_m through the following relationships:

$$\mathbf{H} = -\nabla\varphi_\mathrm{m} \tag{1}$$

$$\nabla\cdot(\mu\nabla\varphi_\mathrm{m}) = 0 \tag{2}$$

where μ is permeability. In a spherical coordinate, the general solutions of the Laplace equation could be expanded by

$$\varphi_\mathrm{m}^i = \sum_{n=1}^{\infty}\left(A_n^i r^n + B_n^i r^{-n-1}\right) P_n(\cos\theta) \tag{3}$$

where i = 1, 2, 3 indicates the regions of background, FM and SC shells, respectively, and $P_n(\cos\theta)$ is the nth order Legendre function. The unknown coefficients A_n^i and B_n^i could be determined from the boundary continuity conditions:

$$\varphi_\mathrm{m}^i|_{r=R_2,R_3} = \varphi_\mathrm{m}^{i+1}|_{r=R_2,R_3} \tag{4a}$$

$$\mu^i\frac{\partial\varphi_\mathrm{m}^i}{\partial r}|_{r=R_2,R_3} = \mu^{i+1}\frac{\partial\varphi_\mathrm{m}^{i+1}}{\partial r}|_{r=R_2,R_3} \tag{4b}$$

$$\varphi_\mathrm{m}^1 = -H_0 r\cos\theta \quad \text{at} \quad r\to\infty \tag{4c}$$

$$B_n^3 = 0 \;\text{ at }\; r = 0 \tag{4d}$$

In Eq. (4c), H_0 denotes the modulus of the uniform incident magnetic field. From these boundary equations, we could obtain the permeability of the FM layer

$$\mu_\mathrm{FM} = \mu^2 = \frac{2\mathrm{R}_3^3+\mathrm{R}_2^3}{2\mathrm{R}_3^3-2\mathrm{R}_2^3}\mu^3 \tag{5}$$

In this work, the background is air (μ^3 = 1) and we take R_3 = 1.5 R_2 = 15 mm, which gives rise to μ_{FM} = 1.631.

Supplementary references

1. Stoppels, D. Developments in soft magnetic power ferrites. *J. Mag. Mag. Mater.* **160**, 323-328 (1996).
2. Souc, J. *et al.* AC magnetic cloak. *New J. Phys.* **15**, 053019 (2013).
3. Solovyov, M., Šouc, J. & Gömöry, F. Magnetic Cloak for low frequency ac magnetic field. *IEEE Trans Appl. Supercond.* **25**, 3 (2015).
